# Insight into Analysis of Essential Oil from *Anisosciadium lanatum* Boiss.—Chemical Composition, Molecular Docking, and Mitigation of Hepg2 Cancer Cells through Apoptotic Markers

**DOI:** 10.3390/plants11010066

**Published:** 2021-12-26

**Authors:** Hany Ezzat Khalil, Hairul-Islam Mohamed Ibrahim, Hossam M. Darrag, Katsuyoshi Matsunami

**Affiliations:** 1 Department of Pharmaceutical Sciences, College of Clinical Pharmacy, King Faisal University, Al-Ahsa 31982, Saudi Arabia; 2 Department of Pharmacognosy, Faculty of Pharmacy, Minia University, Minia 61519, Egypt; 3 Biological Sciences Department, College of Science, King Faisal University, Al-Ahsa 31982, Saudi Arabia; himohamed@kfu.edu.sa; 4 Pondicherry Centre for Biological Sciences and Educational Trust, Kottakuppam 605104, India; 5 Research and Training Station, King Faisal University King Faisal University, Al-Ahsa 31982, Saudi Arabia; hdarag@kfu.edu.sa; 6 Pesticide Chemistry and Technology Department, Faculty of Agriculture, Alexandria University, Alexandria 21545, Egypt; 7 Department of Pharmacognosy, Graduate School of Biomedical & Health Sciences, Hiroshima University, 1-2-3 Kasumi, Minami-ku, Hiroshima 734-8553, Japan; matunami@hiroshima-u.ac.jp

**Keywords:** *Anisosciadium lanatum*, GC-MS, HepG2, BCL-2, CASPASE-3, apoptotic markers

## Abstract

Essential oils have been used in various traditional healing systems since ancient times worldwide, due to their diverse biological activities. Several studies have demonstrated their plethora of biological activities—including anti-cancer activity—in a number of cell lines. *Anisosciadium lanatum* Boiss. is a perennial aromatic herb. Traditionally, it is an edible safe herb with few studies exploring its importance. The current study aims to investigate the chemical composition of essential oil isolated from *Anisosciadium lanatum* using GC-MS, as well as report its anti-cancer potential and its mechanistic effect on HepG2 liver cancer cell lines, and conduct molecular docking studies. To achieve this, the essential oil was isolated using a Clevenger apparatus and analyzed using GC-MS. The cell viability of HepG2 liver cancer and normal fibroblast NIH-3T3 cell lines was assessed by MTT cytotoxicity assay. The effects of the essential oil on cell migration and invasion were assessed using wound healing and matrigel assays, respectively. The effect of the essential oil on migration and apoptotic-regulating mRNA and proteins was quantified using quantitative real-time PCR and Western blot techniques, respectively. Finally, computational docking tools were used to analyze in silico binding of major constituents from the essential oil against apoptotic and migration markers. A total of 38 components were identified and quantified. The essential oil demonstrated regulation of cell proliferation and cell viability in HepG2 liver cancer cells at a sub-lethal dose of 10 to 25 μg/mL, and expressed reductions of migration and invasion. The treatment with essential oil indicated mitigation of cancer activity by aborting the mRNA of pro-apoptotic markers such as BCL-2, CASPASE-3, CYP-1A1, and NFκB. The algorithm-based binding studies demonstrated that eucalyptol, nerol, camphor, and linalool have potent binding towards the anti-apoptotic protein BCL-2. On the other hand, camphor and eucalyptol showed potent binding towards the pro-apoptotic protein CASPASE-3. These findings highlight the effectiveness of the essential oil isolated from *Anisosciadium lanatum* to drive alleviation of HepG2 cancer cell progression by modulating apoptotic markers. Our findings suggest that *Anisosciadium lanatum* could be used as a phytotherapeutic anti-cancer agent, acting through the regulation of apoptotic markers. More well-designed in vivo trials are needed in order to verify the obtained results.

## 1. Introduction

Traditionally, medicinal and aromatic plants have been considered to play essential roles in the field of therapeutics all over the world [1]. Essential oils (Eos), as secondary metabolites produced from such medicinal and aromatic plants, offer great value in terms of their various curative and biological properties. Several investigations have demonstrated the anti-inflammatory, anti-oxidant, anti-fungal, anti-microbial, and cytotoxic activities of such Eos [2,3,4]. Cancer is described as a fatal health condition, considered to be one of major factors leading to death. Cancer affects all human beings and does not differentiate between gender or age, leading to severe negative health and socio-economic impacts. More than 75% of anti-cancer drugs are directly or indirectly derived from medicinal plants [5]. In this context, the discovery of new natural product candidates with anti-cancer properties has unique interest for the purpose of medical care [6]. Many cytotoxic molecules that are of plant origin are widely used in chemotherapy [7]. The contribution of food components to cancer assessment through lifestyle patterns has become popular in diet–disease investigations [8]. Food enriched with vitamins and bioactive phytochemicals could act as tumor-controlling agents to reduce cancer progression, especially in the case of liver or colon cancer [9].

Moreover, a few reports have revealed that Mediterranean EOs and diets based on substances such as vegetables, nuts, whole grains, olive oil, and/or fish oils can reduce cancer-related and total mortality rates [10,11]. Several reports have also investigated the antioxidant activities of essential oils and have shown them to be potent natural sources of antioxidants to control cancer. The abnormal cellular stress causes non-nuclear DNA damage, which leads to inhibition of protein transport and reactive oxygen species formation. The Apiaceae family (formerly Umbelliferae) is one of the families of flowering plants, which consists of 3780 species in 434 genera. It is distributed worldwide [12,13]. Investigations have proven that this family is rich in its diversity of phytochemicals that have been considered as potential sources of new therapeutic drugs, including terpenoids, saponins, flavonoids, coumarins, and poly-acetylenes. In addition, numerous species of the Apiaceae family are reputed to be a significant source of EOs. EOs isolated and identified from this family have been shown to contain more than 760 components in various chemical classes. The identified and reported constituents have shown substantial pharmaceutical and nutritional values [13,14,15,16]. Many members of the Apiaceae family possess various biological activities, such as anti-bacterial, hepatoprotective, cyclo-oxygenase inhibitory, and anti-tumor activities [12]. Most of the members are safe and edible plants. The *Anisosciadium* genus (Apiaceae family) comprises three species—*Anisosciadium isosciadium* Bornm., *Anisosciadium orientale* DC., and *Anisosciadium lanatum* Boiss.—and is endemic to Southwest Asia [17]. Previous investigations have reported on cytotoxicity and antioxidant assessments pertaining to isolated Eos from *Anisosciadium orientale* [17,18]. In this aspect, *Anisosciadium lanatum* Boiss (*A. lanatum*) is a member of the *Anisosciadium* genus that is native and spread wildly throughout the Arabian Peninsula, including Saudi Arabia [19,20]. *A. lanatum* is a perennial herb. Anatomically, its leaves are characterized by a bipinnately parted incision and clasping base. The inflorescences are of a compound umbel type. Its flowers are tiny and demonstrate whitish-pink petals. The bracts are spiny-tipped. The fruitlets of each secondary umbel are aggregated before ripening, but are later separated into spiny units [21,22]. Traditionally, Bedouins have used *A. lanatum* as local medicinal herb: A water extract of the dried aerial parts, including the stem, leaves, flowers, and fruits, is used for skin sores and boils [23]. The young green leaves are a refreshing palatable herb for Bedouin children [24,25]. Additionally, *A. lanatum* has demonstrated veterinary importance, being used in livestock treatment. The extract from the leaves and shoots has been used to treat skin conditions in goats and sheep [19]. Recent studies have reported that *A. lanatum* contains guaiane sesquiterpene and shows anti-proliferative activity towards liver, colon, and lung cells, as well as anti-mutagenic activities [25,26]. A simple study reported an analysis of the EO from *A. lanatum* [20]; however, no previous reports have detailed advanced analyses of EO isolated from of *A. lanatum* and its modulatory cytotoxic effects, including those with respect to cell migration and invasion. In these contexts, the goal of the current exploration is to investigate *A. lanatum*, for the first time, with respect to its suggested potentials.

## 2. Results

### 2.1. Isolation and Identification of Chemical Components of EO

**Figure 1:**
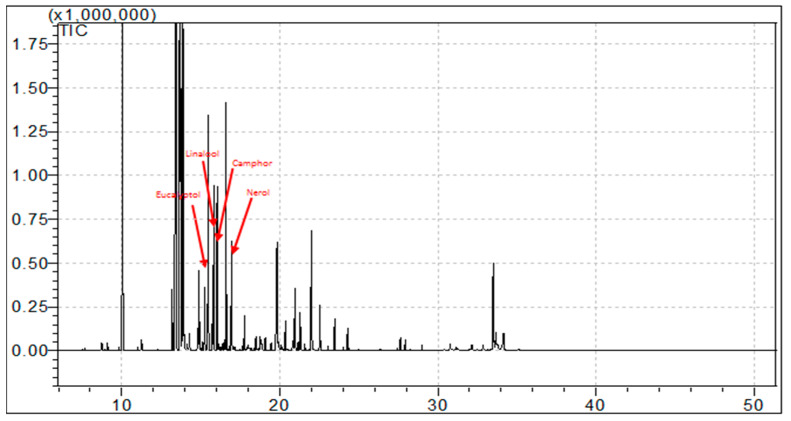
GC-MS chromatogram of EO from *A. lanatum*.

The EO obtained from *A. lanatum* was subjected to detailed gas chromatography–mass spectrometry (GC-MS) analysis (Figure 1). The oil yield was 0.46% volume per dried plant weight. Altogether, 38 components were identified and quantified, corresponding to 94.68% of the total components. The corresponding names of these components are listed in Table 1, according to their elution sequences and retention index (RI). The major identified monoterpene hydrocarbons (10 constituents representing 47.86%) were distributed as follows: α-pinene (10.14%), β-pinene (6.2%), camphene (4.4%), β-myrecene (8.45%), car-4-ene (5.8%), limonene (6.7%), and p-cymene (6.58%). On the other hand, the major identified oxygenated monoterpene components (11 constituents representing 16.44%) were eucalyptol (6.35%), linalool (4.34%), camphor (3.1%), and nerol (4.3%). Regarding the sesquiterpene hydrocarbons (11 constituents representing 22.13%), the results yielded β-farnesene (8.25%), β-caryophyllene (7.25%), and α-humulene (6.45%) whereas the major oxygenated sesquiterpenes (two constituents representing 6.48%) were caryophyllene oxide (7.25%) and α-eudesmol (0.8%). Phenylpropanoids and non-terpene derivatives showed very low percentages.

### 2.2. Proliferation and Cell Viability Assay

**Figure 2:**
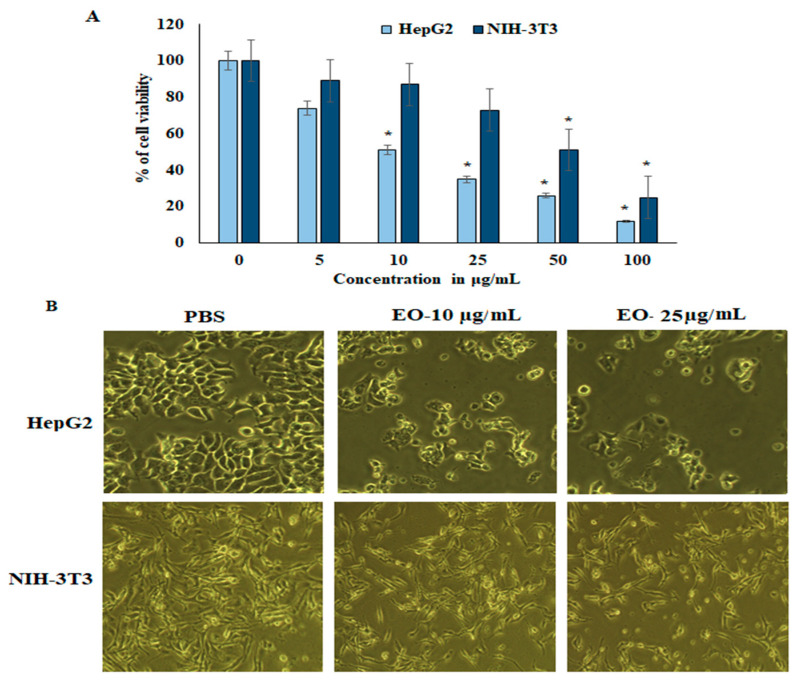
Effect of EO from *A. lanatum* on cell viability. EO inhibited cell growth and cell proliferation in HepG2 liver cancer cells and NIH-3T3 normal murine fibroblast cells: (**A**) HepG2 and NIH-3T3 cell lines were supplemented with EO (0–100 μg/mL) or a vehicle control (0.1% DMSO) for 48 h periods, and cell viability was assessed by MTT assay, and (**B**) HepG2 and NIH-3T3 cells were supplemented with EO (0–100 μg/mL) for 24 h, and cell morphology was examined beneath a phase-contrast microscope. The data are shown as the mean ± SD of triplicate measurements; * *p* < 0.05 when evaluated with respect to control cells.

The suppressive effect of EO was assessed in HepG2 liver cancer cell lines and normal fibroblast NIH-3T3 cell lines. Cell viability and proliferation were determined colorimetrically by 3-(4,5-dimethylthiazol-2-yl)-2,5-diphenyltetrazolium bromide (MTT) assay. HepG2 and NIH-3T3 cell lines were treated with various concentrations (0, 5, 10, 25, 50, and 100 µg/mL) of EO for 24 h. The results revealed that proliferation was potentially inhibited in HepG2 cancer cells in a dose-dependent manner. The IC_50_ of EO in the cell lines was 11.3 µg/mL and 52.1 µg/mL for HepG2 and NIH-3T3, respectively. The sub-lethal dose was considered to be 10–25 µg/mL, showing survival rates of 52.2% and 36.2% in HepG2 and NIH-3T3, respectively. These concentrations were used for further experiments (see Figure 2). On the other hand, NIH-3T3 cell lines showed higher cell proliferation and viability, and it was insignificantly inhibited up to 25 µg/mL of EO. 

### 2.3. Migration (Scratch Wound Assay)

**Figure 3:**
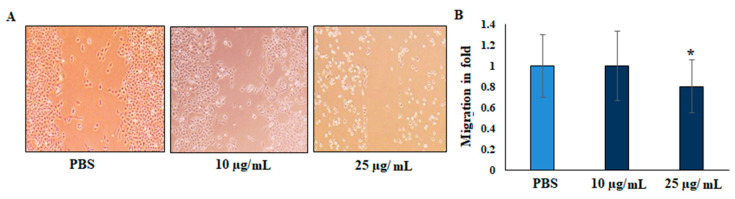
Effect of EO from *A. lanatum* on the migration of HepG2 liver cancer cell lines (scratch wound assay). (**A**) EO inhibits HepG2 human liver cancer cell proliferation and migration. HepG2 cell monolayers were scratched, and cells were supplemented with EO (10 and 25 µg/mL) for 24 h. Migration was determined using an optical microscope (200× magnification) by wound-healing assay. (**B**) Scratch distance was used to calculate the area of the wound and assess wound closure. The data are shown as the mean ± SD of triplicate values; * *p* < 0.05 when evaluated with respect to the control (PBS, phosphate-buffered saline).

The suppressive effect of the EO on migration was evaluated by scratch wound assay. In the scratch mobility assay, HepG2 liver cancer cells were treated with 25 µg/mL of EO supplemented with 1% FBS. The scratch wound was observed to determine the mobility of the cells in the junction, and scratch distance was measured to determine the closure disturbance, compared with that of the untreated control cells. The vehicle-treated HepG2 liver cancer cells significantly migrated after 24 h, whereas a distinct gap remained in the EO-treated groups after 24 h. The gaps were significantly suppressed (0.79 ± 0.05) by EO treatment (Figure 3). To confirm the inhibition effects, a gelatin-coated transwell insert assay was performed for invasion analysis. The relative migration fold of the EO-treated HepG2 liver cancer cells revealed significant differences compared with the untreated cells.

### 2.4. Invasion (Transwell Assay)

**Figure 4:**
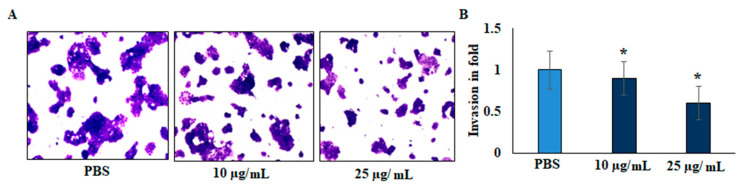
Effect of EO from *A. lanatum* on the inhibition of invasion and angiogenic capacity of HepG2 liver cancer cells. (**A**) EO inhibits human liver cancer cell migration and invasion. HepG2 liver cancer cell lines were supplemented with EO (10 and 25 µg/mL) and loaded to the upper chambers of matrigel-coated transwells. Invasion was determined by total cell counting. Cells invading the lower chamber after 24 h were counted. (**B**) The inhibition percentage of invasion was quantified and expressed relative to the control (untreated cells), whose level of invasion was set at 100%. Invading cells quantified using manual counting and values are noted as fold changes, compared to control. The data are shown as the mean ± SD of triplicate values; * *p* < 0.05 when evaluated against control (PBS, phosphate-buffered saline).

The invasive capacity of cells was evaluated by gelatin-coated transwell assay. As presented in Figure 4, the invasive ability of the EO-treated cells was significantly decreased after 24 h of treatment. Compared with the untreated cells, the relative invasion ratio of EO-treated HepG2 cells was 0.58 ± 0.046. There were significant differences between the treated and untreated cells, and EO significantly decreased the invasive capacity of HepG2 liver cancer cell lines.

### 2.5. Immunoblotting and Localization of Cytochrome-c

**Figure 5:**
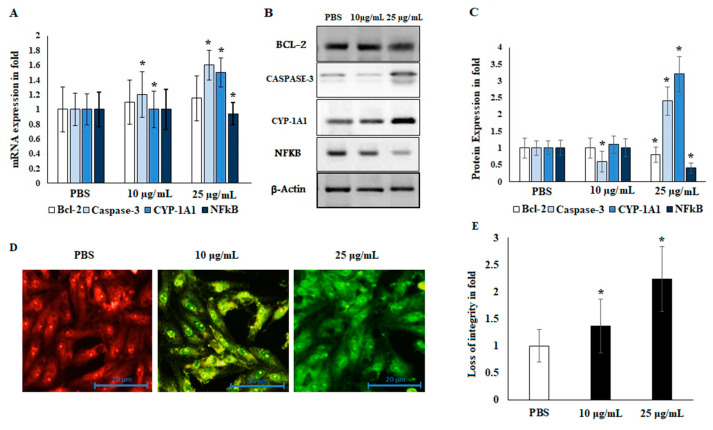
Effects of EO from *A. lanatum* on the mRNA and protein markers of HepG2 liver cancer cells. The effects of EO on the inhibition of apoptotic and angiogenic markers were evaluated by real-time PCR. (**A**) HepG2 liver cancer cells were supplemented with EO (10 and 25 μg/mL). The mRNA of apoptotic and angiogenic markers that was altered in EO-treated cells was quantified using quantitative real-time PCR. GAPDH was used as an internal mRNA control. (**B**,**C**) Alterations in the status of metastasis-associated proteins in response to EO supplementation were inspected using Western blot. HepG2 cells were supplemented with EO (10 and 25 μg/mL) for 24 h. β-actin was utilized as a control. (**D**,**E**) The mitochondrial membrane potential (MMP) was estimated for in EO-treated (10 and 25 μg/mL) HepG2 cell lines after an incubation period of 24 h. The mitochondrial membrane integrity was analyzed using the emission of green fluorescent by ImageJ software. The experimental data are shown as the mean ± SD of triplicate values; * *p* < 0.05 when evaluated against control (PBS; phosphate-buffered saline).

Liver cancer markers are important tools for the evaluation of the migration and invasion of tumor cells. The apoptotic regulatory markers BCL-2 and CASPASE-3 were reciprocally regulated by the EO. Angiogenesis was lost through the regulation of CYP-1A1 and NFκB. EO negatively regulated the NFκB markers and increased CYP-1A1 expression levels in both mRNA and protein markers. These results demonstrate that EO regulates protein expression levels in HepG2 liver cancer cell lines; see Figure 5. The apoptotic marker CASPASE-3 was significantly up-regulated (2.5 ± 0.6-fold; *p* ≤ 0.05), whereas the angiogenic marker NFκB was down-regulated in EO-treated HepG2 cells (0.65 ± 0.1-fold; *p* ≤ 0.05). Furthermore, the metabolic marker CYP-1A1 was significantly up-regulated in EO-treated cells (3.2 ± 0.5-fold; *p* ≤ 0.05). EO-treated HepG2 cells showed a loss of mitochondrial membrane potential (MMP) integrity at both concentrations (10 and 25 μg/mL). Untreated cells showed high MMP integrity by an emission of orange-red fluorescence. On the other hand, apoptotic cells stained with JC-1 showed green fluorescence. Consequently, the EO-treated cells demonstrated an emission of green fluorescence, indicating a loss of mitochondrial membrane integrity and release of the mitochondrial contents, including cytochrome-c (cyt-c), in the cytoplasm (Figure 5D,E). These changes revealed the possible participation of EO in induction of the apoptotic pathway. Therefore, it was suggested that EO treatment (at 25 μg/mL) decreased the migration and invasion abilities of HepG2 liver cancer cells by reciprocal regulation of angiogenesis and apoptotic markers.

### 2.6. In Silico Docking of Major Constituents against BCL-2 and CASPASE-3

**Figure 6:**
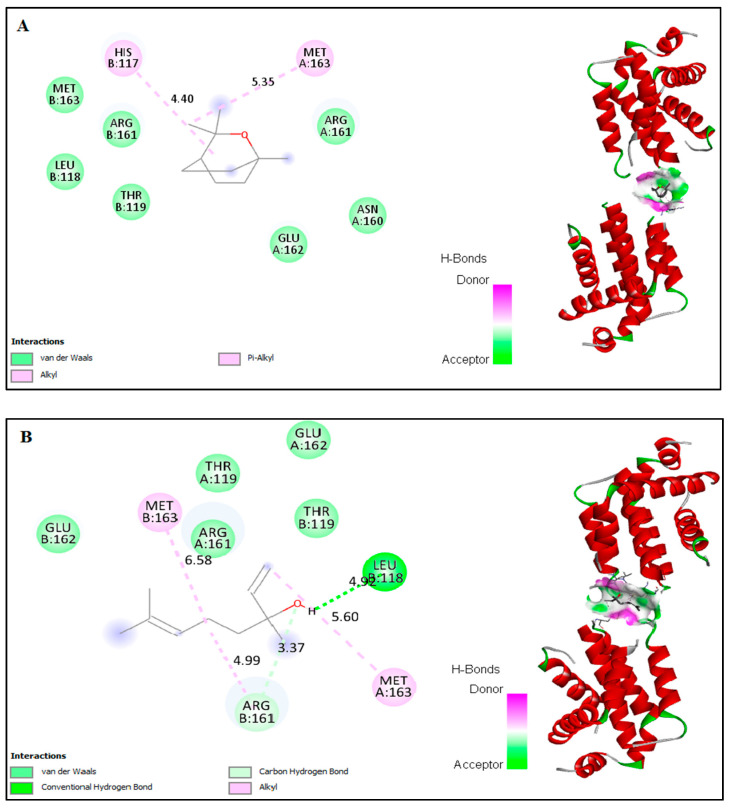

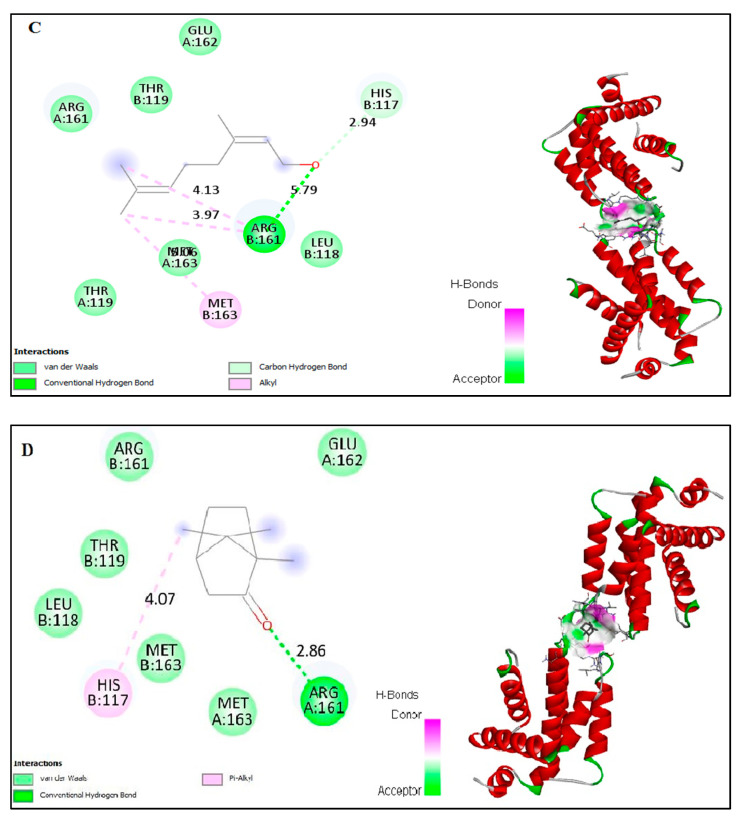
In silico docking of binding interactions of the constituents eucalyptol (**A**), linalool (**B**), nerol (**C**), and camphor (**D**) from EO of *A. lanatum* against anti-apoptotic protein BCL-2. To demonstrate the illustration of interactions in the hydrophobic bond and the other polar bond of BCL-2, we show the amino acid residue analysis of the interacted bond and its length, together with the binding pocket of ligand–receptor interactions.

**Figure 7:**
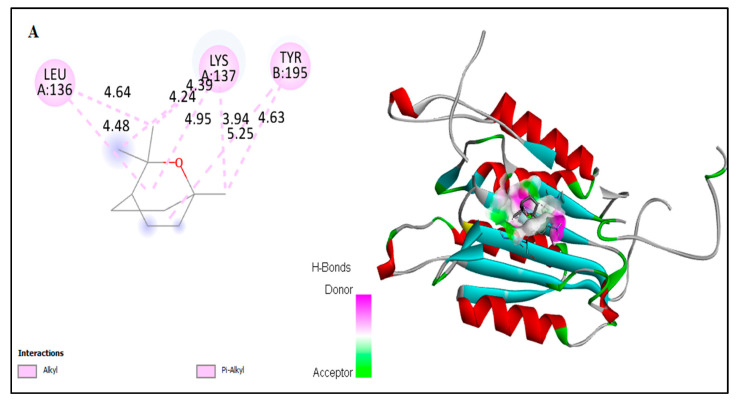

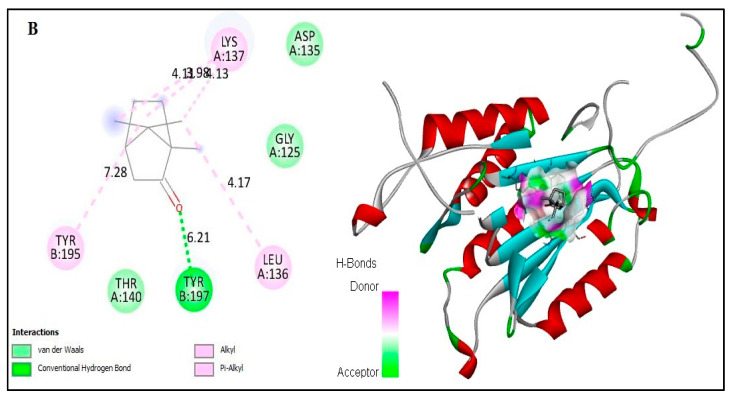
In silico docking of binding interactions of the constituents eucalyptol (**A**) and camphor (**B**) from EO of *A. lanatum* against pro-apoptotic protein CASPASE-3. To demonstrate the illustration of interactions in the hydrophobic bond and the other polar bond of CASPASE-3, we show the amino acid residue analysis of interacted bond and its length, together with the binding pocket of ligand–receptor interactions.

The computational interactions against ligands from the EO with BCL-2 and CASPASE-3 protein receptors were analyzed. In this study, the possible binding patterns and interaction mechanisms of major constituents—including α-pinene, camphene, β-pinene, β-myrcene, car-4-ene, α-terpinene, limonene, p-cymene, β-caryophyllene, α-humulene, β-farnesene, caryophyllene oxide, eucalyptol, linalool, nerol, and camphor—of the EO were analyzed using an auto-docking tool and evaluated using the binding energy and binding efficiency (Figure 6). In general, the binding intermol energy represents the best fit for a ligand in the active site of the target macromolecule. The in silico binding results revealed that, of the major constituents of EO, eucalyptol, linalool, nerol, and camphor showed the highest binding energies and high intermol energies (−3.76, −4.29, −3.37, and −5.5 kcal/mol, respectively). The docking simulation of BCL-2 to EO components resulted in the formation of eight hydrophobic interactions with potent binding energy, ligand efficiency, and intermol energy (Table 2). Several hydrophobic amino acid residues in chain-A and three predominant interactions with chain-B were observed in these EO interaction studies. Four major amino acid residues interacted with EO components in chain-A: Met-163, Arg-161, Lue-136, and Lys-137. Meanwhile, in chain-B, the predominant binding was against His-117, Val- 266, and Thr-266. Interactions against pro-apoptotic CASPASE-3 by the essential oil indicate that eucalyptol and camphor had potent binding compared to the other tested molecules (Figure 7). The binding energies of eucalyptol and camphor were found to be –4.29 and –3.81 kcal/mol, respectively (Table 3). We also observed interactions with the amino acid residues Leu-136 and Lys-137 in chain-A and Thr-195 in chain-B. From the above results, it can be suggested that molecules from EO of *A. lanatum* control liver cancer through apoptotic protein interactions, thus mitigating the migration and angiogenesis of HepG2 liver cancer cell lines.

## 3. Discussion

Plant metabolites exhibit remarkable effects, including anti-cancer and other important biological activities. EOs, among the important secondary plant metabolites, through a long chain of evidence, have been shown to possess several different biological activities [1,27,28,29]. EOs are worth consideration in research in order to highlight their mechanisms of action and pharmacological targets. Chemically, EOs are a complex blend of hydrocarbons and oxygenated hydrocarbons, biosynthesized and arising from the isoprenoid pathways, and mainly consisting of monoterpenes and sesquiterpenes [30]. The EO obtained from *A. lanatum* was analyzed using GC-MS, and interpretation of the analysis revealed and quantified 38 components representing 94.68% of the total components, including monoterpene hydrocarbons (47.86%), oxygenated monoterpenes (16.44%), sesquiterpene hydrocarbons (22.13%), and oxygenated sesquiterpenes (6.48%); see Figure 1 and Table 1. Previous reports have discussed the molecular cytotoxicity effects of EOs, and some examples of isolated compounds from EOs towards various cancer cell lines have indicated the mediation of apoptosis, loss of mitochondrial membrane integrity, and several other mechanisms involving the anti-apoptotic factor BCL-2 and pro-apoptotic protein CASPASE-3 [30,31,32,33]. Camphor and eucalyptol have demonstrated the induction of apoptosis through the down-regulation of anti-apoptotic factor BCL-2 in a human oral epidermoid carcinoma cell line and activation of the CASPASE cascade in oral KB and colorectal cancer cell lines [30,34,35]. Linalool has been shown to lead to a reduction in BCL-2 protein expression in human dermal fibroblast cancer cell lines [36].

Few studies have investigated the anti-cancer activities of EO from *Anisosciadium*. The EO from *A. orientale* has shown antioxidant and anti-cancer activities against various human cancer cell lines [17,18]. In this study, the results from an MTT assay demonstrate that the proliferation of HepG2 was significantly inhibited by the EO of *A. lanatum*. Conversely, NIH-3T3 cell lines showed higher cell proliferation and viability, and were insignificantly inhibited up to 25 µg/mL EO. Considering these results, further studies were carried out on HepG2 liver cancer cell lines (see Figure 2). Cell migratory properties affect tumorigenesis and metastasis. Various studies have targeted cancer cell migration and invasion as potent tools for controlling the progression of cancers [37,38]. In the current study, the cellular functional results revealed that EO (25 μg/mL) from *A. lanatum* exhibited a regulation of cell migration and invasion in HepG2 liver cancer cell lines (Figure 3 and Figure 4). Reliably, the activation of CASPASE-3 and an antagonist of BCL-2 inhibits the potential intrusion of HepG2 liver cancer cells [39]. BCL-2 is an anti-apoptotic protein that controls the release of cytochrome from mitochondria [40]. Mechanistically, the results showed that EO suppressed BCL-2 expression, which could have been reflected in the cell proliferation and survival of HepG2 liver cancer cells. As BCL-2 is a marker for tumorigenesis and neoplastic progression, it may be a potential marker for anti-cancer therapies. Several studies have confirmed that BCL-2 offers potential as a prospective drug target, as it activates the proto-oncogenic effect of the cancer environment [41,42,43]. In this study, EO potentially inhibited the BCL-2 mRNA and protein expression in HepG2 liver cancer cell lines (Figure 5). Many small molecular inhibitors of BCL-2, including ABT-737 and ABT-199, have been investigated extensively [44]. The suppressive effects of BCL-2 and CYP-1A1 are interlinked in cancer prognosis through migratory action and tumorigenesis [45]. Angiogenesis involves regulatory functions played by BCL-2 and CASPASE-3 in reverse roles [38,46]. In this study, the suppression effect of EO on the cell migration process may have been mediated by the suppression of BCL-2 and NFκB markers (Figure 5). The invasiveness activity was also inhibited in EO-treated HepG2 liver cancer cell lines. Blocking of the regulatory role of NFκB suppressed the invasiveness capacity and tumorigenesis in in vitro models. The liver cancer cells were inhibited by EO, which contributed to controlling cancer metastasis [47]. Such suppression regulates the apoptosis (also known as programmed cell death) of tumor cells, morphologically characterized by nuclear damage, chromatin condensation, cell shrinkage, and DNA endonuclease activation with apoptotic bodies [48]. Importantly, in this study, EO induced cell apoptosis in HepG2 liver cancer cells through the activation of mRNA of CASPASE-3 and CYP-1A1 (Figure 5). These genes are apoptotic markers involved in the spreading and invasiveness of cancer cells [44,48,49]. Subsequently high expression of CASPASE-3 and CYP-1A1 could be initiated due to mitochondrial membrane damage. Upon permeabilization of the mitochondrial membrane, caspase activators such as cyt-c were released from mitochondria into the cytosol [50]. The results verify the loss of mitochondrial membrane integrity. Consequently, the mitochondrial contents, including cyt-c, were released into cytoplasm and triggered an intrinsic apoptotic cascade. These findings express the possible activation of apoptosis via cyt-c, which in turn initiates other events in the programmed cell death in EO-treated HepG2 cell lines (Figure-5D, E). Further molecular modulations showed that EO increased the mRNA of CASPASE-3 expression levels, as well as increased and reciprocally regulated the protein expression levels of BCL-2 and NFκB (Figure 5). These results suggest that EO promoted apoptosis in and inhibited the invasiveness of HepG2 liver cancer cells. The previously discussed findings were further confirmed by in silico studies through the potential simulation of anti-proliferative properties of major molecules of EO (Figure 6 and Figure 7). In some studies, 𝛽-sitosterol was shown to have potent binding against apoptotic regulating proteins, whereas isovitexin had the lowest binding affinity detected against BCL-2 and CASPASE-3 proteins [51]. In this study, of the major molecules, only camphor, eucalyptol, nerol, and linalool showed potential binding against BCL-2. Camphor and eucalyptol alone showed significant binding against pro-apoptotic CASPASE-3 regulatory molecules. These findings suggest that the EO from *A. lanatum* can control hepatoma HepG2 liver cancer cells through the reciprocal regulation of apoptotic markers. Taken together, the EO from *A. lanatum* was found to be able to control cancer progression and tumorigenesis, as mediated by modulatory effects on apoptotic markers.

## 4. Materials and Methods

### 4.1. Plant Material 

Aerial parts of *A. lanatum* were collected from Riyadh, Saudi Arabia (April 2014) and were identified by Dr. Engineer Mamdouh Shokry, director of El-Zohria Botanical Garden, Giza, Egypt. A voucher specimen (14-Apr-AL) was deposited at the Herbarium Museum of the College of Clinical Pharmacy, King Faisal University, Al-Ahsa, Saudi Arabia.

### 4.2. Extraction of Essential Oil 

One hundred grams of the carefully collected and air-dried whole *A. lanatum* plant were subjected to hydro-distillation using a Clevenger-type apparatus for 3 h. EO (yield 0.46% volume per dried plant weight) was recovered and dried over anhydrous sodium sulphate. The EO sample was kept in amber-colored vials in a refrigerator at 4 °C until further analyses [4].

### 4.3. Essential Oil Analysis 

The EO was diluted with n-hexane (GC grade, 5 μL:1 mL) and 5 μL were injected into the GC (GC, Model CP-3800, Varian, Walnut Creek, CA, USA) and linked with a mass spectrometer (MS, Model Saturn 2200, Varian, Walnut Creek, CA, USA) equipped with a VF-5ms-fused silica capillary column (5% phenyl-dimethylpolysiloxane, with dimensions of 30 m × 0.25, film thickness was 0.25 μm, Varian). An electron impact (EI) ionization detector was used, with an ionization energy of 70 eV. Carrier gas (helium) was adjusted to have a steady flow rate (1 mL/min). The temperature of the oven was programed as follows: 1, 50, and 5 min at 50, 230, and 290 °C, respectively. The split ratio of injection samples was 1/500, with total time equal to 60 minutes. Identification of the constituents was conducted by comparison of Kovat’s retention indices (RI) relative to a set of co-injected standard hydrocarbons (C10–C28, Sigma-Aldrich, Darmstadt, Germany) [52]. Components were identified by comparing their MS data and their corresponding retention indices with the Wiley Registry of Mass Spectral Data 10^th^ edition (April 2013), the NIST 11 Mass Spectral Library (NIST11/2011/EPA/NIH), and literature data [53]. All of the identified constituents and their relative abundance percentages are listed in Table 1.

### 4.4. Cell Culture and MTT Assay 

Human liver cancer cell lines (HepG2) and a normal fibroblast NIH-3T3 cell line (both cell lines procured from NCCS, Pune, India) were seeded in 96-well plates with a cell population of 1 × 10 ^4^ cells/well in DMEM/F12 with antibiotic solution and 10% FCS (Invitrogen, CA, USA). Cells were incubated in a 5% CO_2_ chamber at 37 °C. The monolayer cultured cells were washed with PBS, and then were treated with EO (10–100 µg/mL) with various test concentrations of tested samples in serum-free media and incubated for 24 h. The medium was aspirated, 0.5 mg/mL of MTT reagent was added, and the solution was incubated at 37 °C for 4 h. After the incubation period, measurements were carried out according to the method protocol described by Khalil et al. (2021) [5].

### 4.5. Migration (Scratch-Wound Assay)

The HepG2 cells were seeded in 6-well plates with a cell population of 1 × 10 ^5^ cells/well in complete DMEM/F12 medium and allowed to attach overnight in a CO_2_ incubator. The media was aspirated with DMEM/F12 with 25% charcoal-stripped FCS for 24 h. After the cells attained confluence, a scratch line was made in the centers of the wells using a sterile tip. The wells were then gently washed with serum-free DMEM/F12 medium and treated again with either DMSO or EO containing DMEM/F12 medium for another 24 h [37]. The scratch-line recovery was recorded using an Optika inverted microscope (200× magnification).

### 4.6. Invasion (Transwell Assay)

The transwell invasion assay was evaluated using a matrigel-coated 12-well Boyden chamber (8 μm PET; Corning, NY, USA), following the methodology described previously by Hanieh et al. (2016) [46], where 4 × 10^4^ cells were cultured in the upper chamber in 600 μL of serum-free DMEM medium with EO. In the lower chamber, 800 μL of DMEM medium with 10% FBS were added.

### 4.7. Immunoblotting

After treatment of HepG2 liver cancer cell lines with different concentrations of EO for 24 h, cells were harvested using trypsin and washed twice with ice-cold PBS. For the immunoblotting analysis, treated cells were lysed in RIPA buffer (Santa Cruz Biotech, CA, USA) for 14 min on ice, then centrifuged at 8000× g for 15 min at 4 °C. Supernatants were collected and estimated using Bradford reagent. An equal amount of protein was denatured at 92 °C for 7 min. Denatured proteins were then separated on SDS-PAGE gels and transferred to PVDF membranes. Membranes were blocked with 5% non-fat milk at room temperature for 30 min, incubated with primary antibodies (BCL-2, 1:1000; CASPASE-3, 1:2000; CYP-1A1, 1:1000; and NFκB, 1:1500) from cell-signaling technology (CST; Beverly, MA, USA) overnight at 4 °C, and then washed and incubated with horseradish peroxidase-conjugated secondary antibody at room temperature for 1 h. Protein bands were visualized by enhanced chemiluminescence (Hisense, Thermo Scientific, Waltham, MA, USA) and analyzed using a Licor analyzer. β-actin (1:2000) was used as an internal control [54].

### 4.8. Mitochondrial Membrane Potential (MMP) Assessment for Localization of Cytochrome-c

The mitochondria-specific fluorescence dye, namely, 5,5’,6,6’-tetrachloro-1,1’3,3’- tetraethyl benzamidazol-carbocyanine iodide (JC-1) (JC-1 MMP assay kit, Abcam), was used to determine the MMP according to the method previously described [55]. HepG2 cells were seeded in 12-well plates and treated with EO (10 and 25 μg/mL) for 24 h. JC-1 working solution was added and incubated at 37 °C for 20 min. Treated cells were washed twice with PBS, replaced with fresh DMEM medium, and captured on a phase contrast inverted fluorescence microscope 200X (Leica 3000 fluorescence microscope). Mitochondrial membrane potentials were monitored by the ratio of red and green fluorescence intensity.

### 4.9. mRNA Expression 

mRNA from treated cells was isolated using Trizol Reagent (Sigma, Darmstadt, Germany) and purified using chloroform and isopropanol. Synthesis of cDNA was carried out using an RT multiscreen kit and amplification was carried out using a thermocycler PCR machine (Eppendorf, Hamburg, Germany). Quantitative analysis of target mRNA such as BCL-2, CASPASE-3, CYP-1A1, and NFkB was performed using a SYBR green master mix (Bio-Rad, California, CA, USA). Details of the primers used for real-time PCR are given in Table 4. Quantification of mRNA expression was achieved using the quant studio software (Bio-Rad, California, CA, USA). Data were normalized to GAPDH levels. All qPCRs were performed at least in triplicate for each experiment [56].

### 4.10. Computational Docking Analysis 

#### 4.10.1. Protein Preparation

For docking studies, three-dimensional structures of anti-apoptotic proteins, such as BCL-2 and CASPASE-3, were retrieved from the RCSB PDB protein data bank [57,58], with their respective PDB ID (4LVT and 3DEK). For docking simulations, water molecules and free hydrogen atoms were removed, and polar molecules were added to all protein structures using the pymol tool and automated AutoDock tool. Further active sites were analyzed in protein receptors using the web-based online tool Q-SiteFinder. All ions, except for the binding site, and non-relevant crystallographic materials were removed. These active sites were chosen as the most favorable binding residues for our docking simulation.

#### 4.10.2. Ligand Preparation

The structures of the major constituents from EO of *A. lanatum*—namely, α-pinene, camphene, β-pinene, β-myrcene, car-4-ene, α-terpinene, limonene, p-cymene, β-caryophyllene, α-humulene, β-farnesene, caryophyllene oxide, camphor, eucalyptol, nerol, and linalool—were retrieved from the Pubchem compound database of the National Center for Biotechnology Information, with the respective IDs, as follows: CID—6654, CID—6616, CID—31253 14896, CID—16211587, CID—7462, CID—22311, CID—7463, CID—5281515, CID—10704181, CID—5281516, CID—1742210, CID—2537, CID—2758, CID—6549, and CID—643820, respectively. The downloaded structures were converted into PDB format using a freely available open-source tool, using pymol and Autodock docking tools.

#### 4.11. Statistical Analysis

Representative data are shown as mean ± standard deviation (*n* = 3) from one experiment. For cell proliferation, migration, invasion, Western blot, and gene expression analyses, samples from the chosen replicate were used. Statistical analysis was conducted by one-way ANOVA followed by Dunnett’s post-hoc test as a reference for comparison to the control group (undifferentiated). All analyses were performed using the GraphPad Prism 7.0 software (GraphPad Software Inc., San Diego, CA, USA), and the statistical results are shown as the corrected p value (* *p* < 0.05).

## 5. Conclusions

In the present study, EO was isolated from *Anisosciadium lanatum* using a hydro-distillation method and its components were identified using GC-MS. The yield of EO was 0.46% *v/w*. In total, 38 components were identified and quantified. The findings suggest that the EO from *A. lanatum* has a controlling effect on hepatoma cells (i.e., the HepG2 liver cancer cell line) through regulation of the apoptotic markers BCL-2 and CASPASE-3. Molecular docking analysis supported the experimental results, which revealed the oxygenated molecules camphor, eucalyptol, nerol, and linalool as having potential virtual binding against the anti-apoptotic marker BCL-2, whereas camphor and eucalyptol alone showed significant binding against the pro-apoptotic regulator CASPASE-3. The obtained results indicate that the EO extracted from *A. lanatum* should be further explored in vivo as a tool for the management of hepatoma cancer diseases.

## Figures and Tables

**Table 1:** Volatile constituents of EO from *A. lanatum*.

No.	Constituents	Rt(min)	RI(Exp)	RI(Lit)	RA%	MS
1	α-pinene	9.03	937	932	10.14 ± 0.6	136.2340
2	camphene	9.29	952	946	4.4 ± 0.1	136.2340
3	β-pinene	9.76	977	977	6.2 ± 0.09	136.2340
4	β-myrcene	9.99	991	988	8.45 ± 0.2	136.2340
5	α-phellandrene	10.28	1005	1002	0.2 ± 0.02	136.2340
6	car-4-ene	13.41	1009	1004	5.8 ± 0.2	136.2340
7	α-terpinene	13.53	1017	1014	3.62 ± 0.07	136.2340
8	limonene	13.74	1030	1224	6.7 ± 0.3	136.2340
9	p-cymene	13.79	1026	1023	6.58 ± 0.1	134.2182
10	terpinolene	15.09	1088	1086	2.07 ± 0.07	136.2340
11	eucalyptol	15.36	1031	1031	6.35 ± 0.2	154.2493
12	linalool	16.37	1099	1095	4.34 ± 0.1	154.2493
13	camphor	16.57	1145	1141	4.3 ± 0.03	152.2334
14	isoborneol	17.03	1167	1165	0.1 ± 0.04	154.2493
15	terpinen-4-ol	17.29	1177	1174	2.4 ± 0.2	154.2493
16	α-terpineol	17.76	1189	1186	1.5 ± 0.05	154.2493
17	fenchyl acetate	17.81	1214	1214	0.1 ± 0.01	196.2860
18	nerol	17.92	1228	1227	3.1 ± 0.1	154.2493
19	bornyl acetate	18.57	1285	1284	0.6 ± 0.04	196.2860
20	methyl geranate	18.61	1321	1319	0.2 ± 0.02	182.2594
21	neryl acetate	18.74	1364	1359	0.1 ± 0.02	196.2860
22	α-copaene	19.56	1376	1374	0.9 ± 0.1	204.3511
23	β-cubebene	19.89	1389	1387	1.3 ± 0.2	204.3511
24	β-caryophyllene	20.45	1424	1424	7.25 ± 0.4	204.3511
25	trans-α-bergamotene	21.63	1435	1432	0.1 ± 0.04	204.3511
26	α-guaiene	21.21	1439	1437	2.02 ± 0.2	204.3511
27	α-humulene	22.69	1455	1452	6.45 ± 0.5	204.3511
28	β-farnesene	22.78	1457	1454	8.25 ± 0.4	204.3511
29	germacrene D	30.98	1481	1484	2.31 ± 0.07	204.3511
30	β-selinene	31.04	1486	1489	0.6 ± 0.03	204.3511
31	β-bisabolene	32.12	1509	1512	0.2 ± 0.03	204.3511
32	γ-cadinene	32.78	1513	1513	0.2 ± 0.04	204.3511
33	caryophyllene oxide	33.86	1640	1638	5.68 ± 0.02	220.3505
34	α-eudesmol	33.94	1653	1652	0.8 ± 0.06	222.3663
35	chavicol	17.73	1256	1247	0.07 ± 0.01	134.1751
36	eugenol	17.92	1357	1356	1.5 ± 0.02	164.2011
37	methyl eugenol	19.94	1406	1402	0.2 ± 0.02	178.2277
38	ethyl isovalerate	8.01	853	856	0.2 ± 0.02	130.1849
**Classes of Constituents**					**RA% (No of Constituents)**	
Total monoterpene hydrocarbons					47.86 (10)	
Total oxygenated monoterpenes					16.44 (11)	
Total sesquiterpene hydrocarbons					22.13 (11)	
Total oxygenated sesquiterpenes					6.48 (2)	
Total phenylpropanoids					1.77 (3)	
Total non-terpene derivatives					0.20 (1)	
Total identified constituents					94.68 (38)	

Values were obtained from three replicates. Mean ± standard deviation (SD) is shown. Rt, retention time; RI (exp), experimental relative retention index; RI (lit), literature relative retention index from MS libraries (Wiley) National Institute of Standards and Technology (NIST); RA, relative abundance; MS, mass spectra values.

**Table 2:** Hydrophobic interaction of potent binding constituents and amino acid residues of target proteins (BCL-2).

Ligand	Eucalyptol	Camphor	Linalool	Nerol
PubChem ID	CID:2758	CID:2537	CID:6549	CID:643820
Binding energy	−3.76	−4.29	−3.37	−5.5
Ligand efficiency	−0.34	−0.39	−0.31	−0.32
Intermol energy	−3.76	−4.29	−4.58	−6.1
Ligand atoms (ring)	Alkyl hydrophobic bond:C9 Pi-alkyl hydrophobic bond:C7	Hydrogen bonds:C2-O Pi-alkyl hydrophobic bond:C8	Alkyl hydrophobic bond:C8, C8 , C1 ,C1 ,C3’-O ,C3’-O Pi-alkyl hydrophobic bond:C5, C3’-O Carbon–hydrogen bond interaction: C3-OH	C-1 C-1-OH C-1 C-1-OH C-1
Docked amino acid residue (bond length)	Chain A: MET`163 (4.75 Å) Chain B: HIS`117 (5.21Å)	Chain A: ARG`161/ HE (2.86 Å) Chain B: HIS`117 (4.07Å)	Chain A: LEU`136 (4.62Å) Chain A: LYS`137 (4.16Å) Chain A: LEU`136 (4.48Å) Chain A: LYS`137 (4.23Å) Chain A: LYS`137/CE Chain B: VAL`266 (3.50 Å) Chain B: TYR`266 (3.50 Å) Chain B: TYR`195 (3.65Å) Chain B: TYR`195 (4.93Å) (3.18Å)	Chain A : MET Chain B : ARG Chain B : HIS

ARG, arginine; HIS, histidine; LEU, leucine; LYS, lysine; MET, methionine; TYR, tyrosine; VAL, valine.

**Table 3:** Hydrophobic interaction of potent binding constituents and amino acid residues of target proteins (CASPASE-3).

Ligand	Camphor	Eucalyptol
PubChem ID	CID_2537	CID_2758
Binding energy	−4.29	−3.81
Ligand efficiency	−0.39	−0.35
Intermol energy	−4.29	−3.81
Ligand atoms (ring)	Hydrogen bonds:C2-O Alkyl hydrophobic bond:C9, C9, O, C8 Pi-alkyl hydrophobic bond: O	Alkyl hydrophobic bond:C7, O, C9, C10, C10, O Pi-alkyl hydrophobic bond:O
Docked amino acid residue (bond length)	Chain A: LEU`136/CG (4.56Å) Chain A: LYS`137/CG (3.88Å) Chain A: LYS`137 (4.38Å) Chain A: LYS`137 (3.95Å) Chain B: TYR`197/ HH (2.04 Å) Chain B: TYR`195 (5.01Å)	Chain A: LYS`137 (4.27Å) Chain A: LYS`137 (4.35Å) Chain A: LEU`136 (4.64Å) Chain A: LEU`136 (4.48Å) Chain B: LYS`137 (3.94 Å) Chain B: LYS`137 (4.95 Å) Chain B: TYR`195 (5.01Å)

LEU, leucine; LYS, lysine; TYR, tyrosine.

**Table 4:** Real-time PCR primer details.

Primer Name	Forward	Reverse	Product Size
BCL-2	TGTGGATGACTGACTACCTGAACC	CAGCCAGGAGAAATCAAACAGAGG	186
CASPASE-3	GTGGAACTGACGATGATATGGC	CGCAAAGTGACTGGATGAACC	212
CYP-1A1	GGCCACTTTGACCCTTACAA	CAGGTAACGGAGGACAGGAA	236
NFκB	TGAAGAGAAGACACTGACCATGGAAA	TGGATAGAGGCTAAGTGTAGACACG	254
β-Actin	AAGATCCTGACCGAGCGTGG	CAGCACTGTGTTGGCATAGAGG	225
